# Effectiveness and Cost-Effectiveness of Repeated Implementation Intention Formation on Adolescent Smoking Initiation: A Cluster Randomized Controlled Trial

**DOI:** 10.1037/ccp0000387

**Published:** 2019-03-07

**Authors:** Mark Conner, Sarah Grogan, Robert West, Ruth Simms-Ellis, Keira Scholtens, Bianca Sykes-Muskett, Lisa Cowap, Rebecca Lawton, Christopher J. Armitage, David Meads, Laetitia Schmitt, Carole Torgerson, Kamran Siddiqi

**Affiliations:** 1 School of Psychology, University of Leeds; 2 Department of Psychology, Manchester Metropolitan University; 3 Institute of Health Sciences, University of Leeds; 4 School of Psychology, University of Leeds; 5 Faculty of Health Sciences, Staffordshire University; 6 School of Psychology, University of Leeds; 7 Faculty of Health Sciences, Staffordshire University; 8 School of Psychology, University of Leeds; 9 Manchester Centre for Health Psychology, University of Manchester; 10 Institute of Health Sciences, University of Leeds; 11 Centre for Health Economics, University of York; 12 School of Education, Durham University; 13 Department of Health Sciences, University of York

**Keywords:** smoking initiation, adolescents, implementation intentions, smoking prevention

## Abstract

***Objective:*** Forming implementation intentions (if–then plans) about how to refuse cigarette offers plus antismoking messages was tested for reducing adolescent smoking. ***Method:*** Cluster randomized controlled trial with schools randomized (1:1) to receive implementation intention intervention and messages targeting not smoking (intervention) or completing homework (control). Adolescents (11–12 years at baseline) formed implementation intentions and read messages on 8 occasions over 4 years meaning masking treatment allocation was not possible. Outcomes were: follow-up (48 months) ever smoking, any smoking in last 30 days, regular smoking, and breath carbon monoxide levels. Analyses excluded baseline ever smokers, controlled for clustering by schools and examined effects of controlling for demographic variables. Economic evaluation (incremental cost effectiveness ratio; ICER) was conducted. Trial is registered (ISRCTN27596806). ***Results:*** Schools were randomly allocated (September–October 2012) to intervention (*n* = 25) or control (*n* = 23). At follow-up, among 6,155 baseline never smokers from 45 retained schools, ever smoking was significantly lower (RR = 0.83, 95% CI [0.71, 0.97], *p* = .016) in intervention (29.3%) compared with control (35.8%) and remained so controlling for demographics. Similar patterns observed for any smoking in last 30 days. Less consistent effects were observed for regular smoking and breath carbon monoxide levels. Economic analysis yielded an ICER of $134 per ever smoker avoided at age 15–16 years. ***Conclusions:*** This pragmatic trial supports the use of repeated implementation intentions about how to refuse the offer of a cigarette plus antismoking messages as an effective and cost-effective intervention to reduce smoking initiation in adolescents.

## What is the public health significance of this article?

This study suggests that getting adolescents to read anti-smoking messages and form implementation intentions about how to refuse the offer of a cigarette in classroom time reduces smoking initiation. Such a classroom-based intervention is an effective and cost-effective way to reduce smoking initiation in adolescents and is readily scalable.

Tobacco smoking continues to be an important cause of morbidity and mortality, particularly later in life (Gowing et al., 2015). Most smokers initiate the habit as adolescents (McRobbie, Bullen, Hartmann-Boyce, & Hajek, 2014; Polosa, Rodu, Caponnetto, Maglia, & Raciti, 2013; Singh et al., 2016) with around 40% of adult smokers having started before they reached 15 or 16 years of age (Warner, 2016). Although quitting smoking at any age is beneficial, maximum health benefit accrues from never initiating smoking. Addiction to nicotine can be established rapidly in adolescence (DiFranza et al., 2007) with strong associations between having a first cigarette (Sargent, Gabrielli, Budney, Soneji, & Wills, 2017) or smoking as infrequently as 1 day in the past month (Saddleson et al., 2016) and progression to regular smoking as an adult. Additionally, early uptake of smoking is associated with more cigarettes smoked (Chassin, Presson, Pitts, & Sherman, 2000; Taioli & Wynder, 1991) and lower quit rates (Ferguson, Bauld, Chesterman, & Judge, 2005) in adulthood. These findings point to the potential value of effective interventions to reduce smoking initiation and avoiding that first cigarette in adolescents. The present article reports a pragmatic trial of an intervention designed to reduce smoking initiation in adolescents by targeting the refusal of offers of a cigarette.

The current intervention was based on implementation intentions. Implementation intentions are specific “if–then” plans (Gollwitzer, 1993). Gollwitzer (1993, 1999) defined an implementation intention as a plan of how, where, and when to perform a behavior. This type of plan establishes a link between a critical situation and a planned behavior (“If I encounter Situation X then I will do Y”). Through forming an implementation intention, it has been argued that an individual passes control of goal directed activities from the self to critical situations (e.g., Aarts, Dijksterhuis, & Midden, 1999). The critical situation when encountered then prompts the intended behavior, through automatic activation of the plan (see Webb & Sheeran, 2003). In this way implementation intentions facilitate quick and reliable initiation of the intended behavior by increasing readiness to respond to specified opportunities (when “X” occurs; Gollwitzer, 1993).

Implementation intentions have been found to be effective means to change a range of behaviors (Gollwitzer & Sheeran, 2006), including promoting smoking cessation (Armitage, 2016). Empirical findings indicate that the effects of forming implementation intentions are often contingent on the presence of strong motivation or goal intention to perform the behavior (e.g., Prestwich, Sheeran, Webb, & Gollwitzer, 2015; Sheeran, Webb, & Gollwitzer, 2005). A number of studies (including the present one) therefore use interventions that combine the formation of implementation intentions with the presence of motivational messages about the target behavior (e.g., Prestwich, Lawton, & Conner, 2003).

Two previous studies tested implementation intentions in conjunction with antismoking messages in relation to smoking initiation in adolescents. In a pilot study, Higgins and Conner (2003) tested the effects of engaging with antismoking messages plus forming a single implementation intention on self-reported ever-smoking 2 months later. Implementation intentions were formed in relation to refusing offers of a cigarette (intervention; e.g., If offered a cigarette then I will say “no thanks, I do not smoke”) or completing homework (control). Of the 104 baseline never smokers, 0% initiated smoking in the intervention group, while 6% initiated smoking in the control group. In a later explanatory trial with 1,338 adolescents, Conner and Higgins (2010) tested the effects of forming implementation intentions on how to refuse the offer of a cigarette after engaging with antismoking messages on eight occasions (intervention). The control conditions also included engaging with antismoking messages on eight occasions plus an intervention designed to promote self-efficacy not to smoke, or forming implementation intentions about completing homework or promoting self-efficacy to complete homework. Compared with the combined control conditions, the intervention was shown to reduce self-reported smoking and breath carbon monoxide levels significantly at 4 years postbaseline.

The present research was designed as a pragmatic cluster randomized controlled trial of the effectiveness of forming repeated implementation intentions about how to refuse an offer of a cigarette after engaging with antismoking messages compared to usual practice on tobacco control. Previous studies (Conner & Higgins, 2010; Higgins & Conner, 2003) showed the efficacy of combining implementation intentions with antismoking messages compared with antismoking messages alone. Therefore, this pragmatic trial compared the combined intervention with a control condition using the same intervention techniques (i.e., implementation intentions combined with persuasive messages) but focusing on a distinct behavior (completing homework) rather than, for example, comparison with antismoking messages alone. In the control condition used here adolescents formed repeated implementation intentions about how to complete homework after engaging with prohomework messages.

This present intervention (i.e., forming repeated implementation intentions on how to refuse the offer of a cigarette after engaging with antismoking messages), if shown to be effective in a pragmatic trial, could be deployed across schools to reach the majority of adolescents in order to tackle smoking initiation in this age group. In addition, this intervention is relatively low cost, requiring only 30–50 min per session for teachers to implement in classroom time (including engaging with antismoking messages and completing an implementation intention questionnaire; Conner & Higgins, 2010; Higgins & Conner, 2003). This contrasts with other antismoking interventions tested in this age group that tend either to have only mixed evidence for their effectiveness (for reviews see MacArthur, Harrison, Caldwell, Hickman, & Campbell, 2016; Thomas, McLellan, & Perera, 2015; Wiehe, Garrison, Christakis, Ebel, & Rivara, 2005), or have effectiveness evidence but are high cost (Campbell et al., 2008; Peterson, Kealey, Mann, Marek, & Sarason, 2000).

## Method

### Study Design and Participants

Secondary schools in two areas of England (Leeds and Staffordshire Local Education Authorities) were eligible for inclusion in the study. Head teachers provided written consent that their schools would participate in the trial and continue usual smoking education and policies on tobacco control for the trial duration. Schools sought parental consent (i.e., passive consent) by writing to parents of pupils in the relevant year group (Year 7 at baseline, 11- to 12-year-olds). Very few parents asked for their child to be excluded from data collection sessions. All adolescents in the relevant year group were eligible for participation. Adolescents provided active assent by completing questionnaires. As passive consent was used, ethical/governance procedures required that adolescent data be collected anonymously and so matching of data across time points was based on individually generated codes.

The University of Leeds (School of Psychology, Faculty of Medicine and Health) ethical review committee approved the study (reference 12–0155 on September 24, 2012). The study was registered on October 26, 2012 (ISRCTN27596806) before any intervention sessions. There were no changes to the methods after trial commencement. Details of the trial protocol have been published previously (Conner et al., 2013).

### Randomization and Masking

School was the unit of randomization. Schools were randomized by random number generator to intervention or control conditions on a 1:1 ratio by the trial statistician (RW). Randomization took place before recruitment of participants within each school. Due to the nature of the intervention, adolescents, teachers administering the intervention, heads of school, and data collection assistants were aware of group allocation. The trial statistician who conducted the analyses was initially blinded to condition.

### Procedures

Self-reported data were collected at baseline plus 12, 24, 36, and 48 months postbaseline by research staff (present to answer questions) via questionnaire in groups (classes or year group assemblies) with adolescents requested not to confer. At each time point a smokerlyzer measure of breath carbon monoxide levels was taken individually with readings not available to adolescents.

The eight intervention sessions took place separately to data collection in classroom time (with each session containing approximately 26 adolescents) approximately every 6 months starting within 2 months of baseline data collection and were each led by a teacher. The content of sessions was designed to be matched (in relation to duration and frequency plus the use of written motivational materials and an implementation intention formation task) across the two conditions but focusing on smoking (intervention condition) or completing homework (control condition) as an unrelated behavior. Adolescents engaged with motivational materials (read antismoking messages or prohomework messages plus engaged in related tasks designed to increase engagement with the messages) and then completed implementation intentions sheets in relation to the target behavior (not smoking in intervention condition; completing homework in control condition). The target behavior in the control condition (completing homework) was selected to be a nonhealth related behavior appropriate for adolescents. The interventions were designed to run within a standard classroom session (50 min) with the majority (60%) of the time devoted to the messages.

Implementation intention formation was consistent across intervention sessions. Adolescents were first required to tick an option to indicate how they could refuse smoking this school term (“Tick ONE of the following things you could say if you were offered a cigarette or if you were tempted to smoke . . .; No thanks, smoking makes you smell awful; No, I do not want yellow teeth; No, I do not want to get addicted; No thanks, if you’re buying cigarettes you’re buying cancer; No it’s really bad for my asthma”). They were then requested to write in the selected response or generate a new response of their own to complete a statement (“If someone offers me a cigarette, then I will say . . .; e.g., No cancer sticks for me”). Adolescents were then required to indicate *where* they would not smoke (“Tick ALL the places where you will not smoke: I will not smoke at school; I will not smoke at home; I will not smoke at a party; I will not smoke with my friends; I will not smoke if I’m offered a cigarette”) and to respond to a question about smoking this school term (“I think I can make sure I do not smoke this term: yes, no”). The task was similar in the control condition but completed in relation to completing homework. Participants completed the implementation intention task individually by ticking boxes and writing down responses. The implementation intention sheets were collected in by the teacher and returned to the research team.

The motivational materials provided antismoking or prohomework messages and were paper based. The motivational materials were different in each session (i.e., eight sets of materials), were all judged to be age-appropriate by an experienced school teacher, and were similar in content to that used in our previous work (Conner & Higgins, 2010; Higgins & Conner, 2003). For example, the first set of antismoking materials (“Smoking: It’s not worth it”) focused on 10 reasons not to smoke and included text and pictures along with a quiz designed to promote engagement with the materials. Full copies of the implementation intention sheets and motivational materials can be obtained from the first author.

Training sessions were run with teachers in each year of the study. These were 45-min sessions run in each school that focused on the broad purpose of the intervention and details of the intervention content (motivational messages and implementation intention sheets plus a plan of how to run the session). An opportunity to discuss the content and any potential problems with delivery was provided. The need to stick to the planned content and ensure all implementation intention sheets were fully completed was emphasized. A teacher in each school acted as a coordinator and monitored the delivery of all sessions and was available to answer teachers’ questions.

### Outcome Measures

Four measures of smoking were used as outcomes at the 48-month follow-up. Self-reported cigarette use was assessed at each time point using a standardized measure (Office for National Statistics, 1997); adolescents ticked one of: (a) I have never smoked; (b) I have only tried smoking once; (c) I used to smoke sometimes, but I never smoke cigarettes now; (d) I sometimes smoke cigarettes now, but I do not smoke as many as one a week; (e) I usually smoke between one and six cigarettes a week; (f) I usually smoke more than six cigarettes a week. This was used to create our first two measures of smoking: *ever smoking* (ticking response a coded 0; ticking responses b–f coded 1); *regular smoking* (ticking responses a–d coded 0; ticking responses e–f coded 1).

Any smoking (last 30 days) was assessed at 48-month postbaseline only (self-reported number of days in last 30 days using each of cigarettes, cigars, pipes, or sheesha/hookah was recorded and summed). Any smoking (last 30 days; 0 days coded 0; ≥1 day coded 1) was our third smoking measure.

Breath carbon monoxide (CO) levels (in parts per million; COppm) were assessed using the Micro + Smokerlyzer® CO Monitor (Bedfont Scientific Limited, Kent, United Kingdom) at each time point. However, the short half-life (four-six hours) of breath CO means that such measures are only reliable and valid for assessing recent cigarette smoking (Bedfont, 2017; Jarvis, Tunstall-Pedoe, Feyerabend, Vesey, & Saloojee, 1987; Stookey, Katz, Olson, Drook, & Cohen, 1987). A variety of cut-offs have been used in the literature to indicate smoking in adults. We used the cut-off recommended by the device manufacturer as a clear indication of recent smoking in adolescents (≤6 ppm CO coded as 0; >6 ppm CO coded as 1). Breath CO >6 ppm was our fourth smoking outcome measure.

For the three smoking measures taken at each time point (ever smoking, regular smoking, breath CO >6 ppm) we also created measures of smoking across Time Points 2 to 5 (based on being categorized as smoking on a measure on at least one of the time points).

### Other Measures

Other measures were assessed as covariates and/or moderators and measured at 48 months follow-up. At the school level we recorded geographical area (Leeds; Staffordshire) and size (number of pupils), and area level socioeconomic status (percentage of pupils in a school receiving free school meals; Croxford, 2000). At the individual level we assessed gender, ethnicity (self-reported classification dichotomized into non-White vs. White) and individual-level socioeconomic status (four-item Family Affluence Scale [FAS] scored 0–9 with higher scores indicating greater affluence; Boyce, Torsheim, Currie, & Zambon, 2006).

Fidelity checks assessed adherence, quality of delivery, and exposure to the intervention. The study coordinator in each school was requested to monitor adherence and provide feedback on the number of intervention sessions in their school not run as planned. Teachers were requested to return to the study coordinator completed implementation intention sheets after each session. These were subsequently collected from each school. For approximately half of these sessions, teachers were also requested to complete feedback sheets on session delivery. The feedback sheets included a rating of how well the session went (“The lesson went incredibly well;” *strongly disagree*, *disagree*, *neither agree nor disagree*, *agree*, *strongly agree*). Quality of delivery was also assessed in observation of sessions by researchers. Approximately 7% of sessions were observed by researchers, including at least one session in each school. Observation sheets included a rating of overall quality of delivery (“Overall session quality was . . .;” *low*, *moderate*, *satisfactory*, *good*, *high*). Exposure to the intervention was assessed by self-reported questions from participants at the final follow-up. Those in the intervention (antismoking) condition were asked to indicate which sessions they attended by checking a box next to each session (identified by number, short title, and image of the antismoking information) to give a score between 0 and 8. All participants were requested to indicate if they had moved school since the beginning of the study and to specify the old school and year of change (coded into total numbers changing school, numbers moving between schools in different conditions, numbers moving from nonstudy schools or nonspecified schools).

Data relevant to costing the intervention fully were also collected. A number of other measures were taken but are not reported here (full details available from first author along with intervention materials, analysis scripts, and raw data).

### Statistical Analyses

Based on a power of 90% to detect a 5% difference in smoking rates, an intraclass correlation (ICC) of .01, and alpha of .05, prior sample size calculations indicated the need for at least 3,672 adolescents from 36 schools in the analyses (Conner et al., 2013). We first summarized the measures taken for the full sample and the intervention and control conditions. The main analyses tested for differences between the intervention and control conditions at 48-month postbaseline in each of the four smoking measures among those who were self-reported never smokers at baseline. Those who self-reported ever smoking at baseline (*N* = 301) were removed from all analyses. The largest amount of missing data was for baseline ever smoking, principally due to a failure to match individually generated codes. Missing self-reported ever smoking at baseline was imputed to be zero (i.e., never smoking). Missing data from other variables ranged from 0.2% for gender to 5.8% for any smoking (in last 30 days; see Table 1 for details of numbers of missing data points for each variable) and only 88% of the 6,115 never smokers in the sample would have been available for analysis under the traditional listwise deletion method across these variables. Data were primarily missing due to item nonresponse. We addressed the problem of missing data through multiple imputation using chained equations (MICE; van Buuren & Groothuis-Oudshoorn, 2011) after confirming that the missing values were missing at random. The mice command in R was used to generate 20 imputed data sets that were analyzed using the pooled command. Imputed values compared reasonably with observed values and the results using listwise deletion were similar to multiple imputation, so imputed results are presented.[Table-anchor tbl1]

Based on the distribution and frequency of outcomes, log binomial regressions, implemented in R were used to predict each smoking outcome (ever smoking; any smoking in the last 30 days, regular smoking, breath CO >6 ppm) controlling for the clustering among schools (multilevel modeling). Condition and percentage free school meals were Level 2 variables in these models, while gender, ethnicity, and the FAS scores were Level 1 variables. We report the risk ratio (RR), the 95% confidence interval around the risk ratio (95% CI), and the *p* value for each predictor variable in these regressions. The RR is the ratio of likelihood of the outcome (in this case smoking) across the compared conditions (intervention vs. control). For each step we also report the ICC. At Step 1 condition was entered, while at Step 2 we examined the effects of controlling for demographic variables (school SES; boys vs. girls; non-White vs. White ethnicity; individual level of socioeconomic status based on FAS). At Step 3 we tested whether each of these demographic variables significantly moderated the effects of the intervention. For outcome measures taken at each of the postbaseline time points (ever smoking, regular smoking, and breath CO >6 ppm), sensitivity analyses assessed intervention effects on smoking on at least one time point (i.e., for each smoking measure an outcome was created: 0 = not smoking at any time point; 1 = smoking at one or more time points). Fidelity analyses also examined whether attending no smoking intervention sessions versus a few or most smoking intervention sessions influenced the key findings. Fidelity analyses also examined whether the key findings were influenced by excluding participants who self-reported changing school.

The economic evaluation was based on the incremental cost of the intervention per averted smoker at age 15–16 years. The costs of implementing the intervention were gathered by researchers during the study and expressed in United Kingdom sterling in 2017 prices (converted to U.S. dollars) based on wages and transport costs as at August 2017 provided by the Office for National Statistics. Costs included intervention development (printing material), delivery (travel and time incurred in providing training and support), and receipt (teacher time in undertaking training). Costs over the 4-year period were discounted at 3.5% per annum consistent with NICE guidelines (National Institute for Health & Care Excellence, 2018). An incremental cost-effectiveness ratio (ICER) was calculated based on the incremental cost per adolescent of implementing the intervention divided by the difference in the proportion not smoking across conditions.

## Results

### Sample Description

The study took place between September 2012 and January 2017. A total of 73 secondary schools were eligible for inclusion in the study. Of these, 48 schools agreed to participate and were randomized to intervention (*n* = 25) or control (*n* = 23) conditions. Three schools subsequently withdrew from the study before data collection began because of changes in decisions by school management and also declined when requested to participate at the final time point. The remaining schools (25 intervention, 20 control) were retained for the duration of the 4-year trial.

Table 1 provides details of the sample overall and by condition for school and individual level data (and numbers of missing data points). At the school level, the intervention and control conditions were not significantly different in terms of school size or geographical area (neither had effects on the results and are not considered further here), nor in terms of percentage of pupils eligible for free school meals. Compared with the value for the United Kingdom as a whole (*M* = 13.8; Department for Education, 2017), our 45 schools had a slightly higher percentage of pupils eligible for free school meals (*t*_*one sample*_(44) = 1.98, *p* = .054). This was also true for free school meals data in our schools from each of the two geographical areas compared with appropriate regional data (Leeds, *M* = 20.63, *SD* = 11.13 vs. *M* = 16.5 for area, *t*_*one sample*_(19) = 1.66, *p* = .114; Staffordshire, *M* = 13.28, *SD* = 5.97 vs. *M* = 9.30 for area, *t*_*one sample*_(24) = 3.34, *p* = .003). This indicates that the included schools were slightly more deprived than comparable schools.

**Figure 1 fig1:**
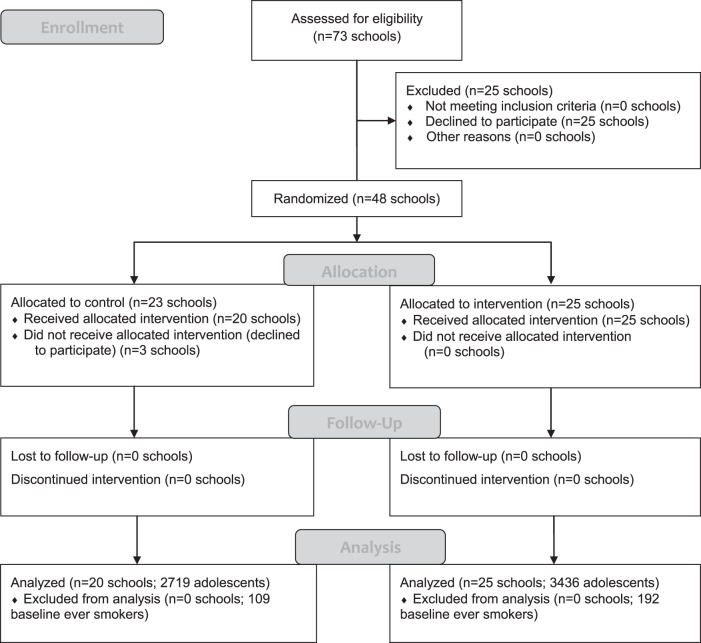
Flow diagram of clusters and individuals through phases of randomized trial.

At the individual level, the baseline sample did differ in ever smoking by condition, with a significantly higher proportion of ever smokers in the intervention condition. Across the 45 included schools, at 48-month follow-up, data were available from 6,155 adolescents aged 15–16 years who did not smoke at baseline (i.e., 301 baseline ever smokers excluded; see Figure 1). The 48-month follow-up sample was not significantly different between control and intervention conditions in proportion of boys or White ethnic background and did not differ in mean FAS scores (see Table 1). Follow-up self-reported *ever smoking* and *any smoking (last 30 days*) were significantly lower in the intervention compared to the control condition. In contrast, although *regular smoking* was lower in the intervention compared to the control conditions, this difference did not approach statistical significance. *Breath CO* >6 ppm rates were low in both conditions, but significantly lower in the intervention compared to the control condition (see Table 1).[Fig-anchor fig1]

### Predicting Smoking Outcomes

Log binomial regressions in a multilevel model that controlled for the effects of school (and excluded baseline ever smokers) showed that self-reported *ever smoking* was significantly lower in the intervention compared to the control condition (Model 1, Table 2). This effect for condition on *ever smoking* remained significant when also controlling for demographic variables (Model 2, Table 2). Free school meals, gender, White ethnicity, and FAS were also significant predictors of *ever smoking* at this step, with higher levels of *ever smoking* being associated with more free school meals, being female, being White and with lower family affluence. None of the demographic covariates significantly moderated the effects of the intervention on *ever smoking* (free school meals: RR = 1.00, *p* = .790; gender: RR = 1.07, *p* = .387; ethnicity: RR = 1.14, *p* = .246; family affluence scale: RR = 1.02, *p* = .402). Sensitivity analyses also showed that the effect of the intervention on reducing *ever smoking at any time point* was significant when controlling for covariates (*p* = .003) or not (*p* = .016; see supplementary Table 1).[Table-anchor tbl2]

A similar pattern was apparent for *any smoking (last 30 days*) with the condition effect being significant (i.e., less smoking in the intervention condition) when not controlling (Model 1, Table 2) or controlling (Model 2, Table 2) for demographic variables. Lower family affluence (FAS) was significantly associated with higher levels of *any smoking (last 30 days*). None of the demographic covariates significantly moderated the effects of the intervention on *any smoking (last 30 days*; free school meals: RR = 1.00, *p* = .851; gender: RR = 1.04, *p* = .732; ethnicity: RR = 1.25, *p* = .177; family affluence: RR = 0.98, *p* = .513).

The intervention had a weaker effect in relation to *regular smoking* and *breath CO* >6 ppm. Although *regular smoking* was lower in the intervention compared with the control condition (see Table 1), this difference was not statistically significant (Model 1, Table 2). The condition effect remained nonsignificant (*p* = .092) when also controlling for demographic variables (Model 2, Table 2). More *regular smoking* was significantly associated with being in the control compared to the intervention condition, more free school meals, being White, and being from a less affluent family (FAS). None of the demographic covariates significantly moderated the effects of the intervention on *regular smoking* (free school meals: RR = 1.00, *p* = .945; gender: RR = 1.23, *p* = .283; ethnicity: RR = 1.47, *p* = .197; family affluence: RR = 0.91, *p* = .102). Sensitivity analyses showed that the effect of the intervention on reducing *regular smoking at any time point* was significant when controlling for demographic covariates (*p* = .036) but was not significant when not controlling for covariates (*p* = .156; see supplementary Table 1). Similarly, *breath CO* >6 ppm was lower in the intervention compared to the control condition (Model 1, Table 2), although this difference was not statistically significant (*p* = .066). The effect for condition on *breath CO* >6 ppm became significant (*p* = .024) when also controlling for demographic variables (Model 2, Table 2). Lower family affluence scores (FAS) were also significantly related to higher levels of *breath CO* >6 ppm. None of the demographic covariates significantly moderated the effects of the intervention on *breath CO* >6 ppm (free school meals: RR = 0.92, *p* = .061; gender: RR = 0.86, *p* = .689; ethnicity: RR = 1.71, *p* = .358; family affluence: RR = 0.86, *p* = .149). Sensitivity analyses showed that the effect of the intervention on *breath CO* >6 ppm at any time point was not significant when controlling for covariates (*p* = .079) but was significant when not controlling for demographic covariates (*p* = .027; see supplementary Table 1).

### Fidelity Analyses

School coordinators informed us about a total of 37 sessions (approximately 1.6%) across control and intervention conditions that did not run as planned. We received teacher feedback on 797 (88%) out of 905 sessions from teachers, with 11 (1%) responding *strongly disagree*, 69 (7%) *disagree*, 348 (44%) *neutral*, 337 (42%) *agree*, and 30 (4%) *strongly agree* to the statement that the “lesson went incredibly well.” A total of 73 individual sessions (30 control, 43 intervention) were observed and scored for quality. In relation to overall session quality, none were rated as *unsatisfactory*, four (5%) as *moderate*, 16 (22%) as *satisfactory*, 46 (63%) as *good*, and seven (10%) as *high quality*. Lower ratings were mainly attributable to disruptive student behavior impacting on learning, too large a group to process the activities interactively, insufficient time to complete the activities, or insufficient staff input (e.g., no exploration of the activities as a group/students completed independently).

Completed implementation intention sheets were returned for approximately 91% of adolescents (89% in intervention; 95% in control) and the vast majority of sheets were scored as complete (88%) with no difference between conditions (87% in intervention; 90% in control). It was not possible to match individually generated codes to data on smoking thus precluding an analysis of the impact of completion on intervention effectiveness.

In the intervention condition at the final time point, a total of 496 (14%) participants reported attending no (0) intervention sessions, 542 (15%) reported attending a few (one to four) intervention sessions, and 2,590 (71%) participants reported attending most (five to eight) intervention sessions. Analyses indicated that controlling for clustering by schools and covariates there were generally few differences between those attending no intervention sessions and those who attended a few intervention sessions for each of the smoking outcomes (*ever smoking*: RR = 1.11, 95% CI [0.96, 1.29], *p* = .143; *any smoking (last 30 days*): RR = 0.78, 95% CI [0.62, 0.98], *p* = .032; *regular* smoking: RR = 0.96, 95% CI [0.69, 1.34], *p* = .811; *breath CO* >6 ppm: RR = 0.92, 95% CI [0.48, 1.76], *p* = .794). In contrast, there were significantly lower rates of smoking in those who attended most intervention sessions compared with those attending no intervention sessions for each of the smoking outcomes (*ever smoking*: RR = 0.76, 95% CI [0.68, 0.86], *p* < .001; *any smoking (last 30 days*): RR = 0.61, 95% CI [0.52, 0.72], *p* < .001; *regular smoking*: RR = 0.57, 95% CI [0.45, 0.73], *p* < .001; *breath CO* >6 ppm: RR = 0.41, 95% CI [0.24, 0.70], *p* = .001).

A total of 540 (9%) participants reported a change of school within the study period. A total of 82 moved between schools in the same condition, 86 moved between schools in different conditions, and 372 moved in to the study from nonstudy schools or did not specify the school they had moved from. These numbers were similar for the control and intervention conditions. Sensitivity analyses indicated that excluding these 540 participants did not substantively alter the main findings (i.e., no change in significance of condition for any smoking outcomes).

### Economic Analyses

The intervention was costed at $1,391 (£1,031) per school over the 4-year program duration. Twenty-eight percent of the intervention cost was associated with covering teacher time (seven members per school) to attend an intervention training session (45-min duration) pertaining to how to deliver the intervention, conservatively assuming that: (a) teachers attend the training every year, and (b) their workload is already fully allocated. The rest of the intervention cost stemmed from printing and delivering material to schools, researcher travel, and time incurred in training teachers and various administrative support tasks.

Based on an average school size of 160 pupils (initially aged 12–13 years), the intervention cost is $8.69 (£6.44) per adolescent (see supplementary Table 2 for further cost details). When comparing this intervention cost to the trial arm difference in the proportion who were never smoking at follow-up (6.5% more on *ever smoking* measure in intervention arm; Table 1), the intervention yields an incremental cost-effectiveness ratio (ICER) of $134 (£99) per ever smoker avoided at age 15–16 years. A sensitivity analysis including the sunk cost of designing the intervention ($21,159 [£15,680]) and assuming teachers’ delivery of sessions requires extra time (i.e., they are done outside existing sessions), the intervention cost increases to $18.99 (£13.33) per adolescent. This yields an ICER of $292 (£205) per smoker avoided at age 15–16 years based on *ever smoking* assessment.

## Discussion

The current study shows that the repeated formation of implementation intentions about how to refuse offers of a cigarette alongside motivational antismoking messages were effective in significantly reducing *ever smoking* (6.5% reduction) and *any smoking (last 30 days*; 4.8% reduction). The intervention may also provide a cost-effective means to reduce smoking initiation in adolescents. The evidence in relation to *breath CO* >6 ppm and particularly *regular smoking* was less consistent, with a significant effect for the former only when controlling for demographic variables and a nonsignificant effect for the latter (controlling for demographic variables or not). None of the intervention effects for the four smoking variables were significantly different in schools serving more or less deprived areas, in boys versus girls, in non-White versus White adolescents, or at different levels of family affluence. Sensitivity analyses showed that a similar pattern of findings was apparent when examining *ever smoking*, *regular smoking*, or *breath CO* >6 ppm across any of the four time points postbaseline. This suggests that the current findings were not restricted to smoking outcomes at the final data collection point.

Relatedly our fidelity analyses suggest that the majority of intervention sessions were completed as planned. We were unable to examine the impact of completing the implementation intention sheets on outcomes due to problems with matching individual codes, nevertheless a high percentage of sheets were completed appropriately. We were, however, able to assess the impact of the number of smoking sessions attended. This indicated that for each of the four smoking outcomes significant effects were mainly associated with attending between five and eight intervention sessions. This contrasts with Conner and Higgins (2010) who did not find number of sessions attended influenced the effectiveness of the intervention in reducing smoking initiation, although over 90% of participants in that study attended between three and eight intervention sessions. Together the two studies might suggest the value of multiple implementation intention sessions over single sessions in reducing smoking in adolescents, although adolescents may not need to attend all sessions to benefit. Our fidelity analyses also indicated our findings were not influenced by participants moving between schools during the study.

The mixed findings for *regular smoking* are worth further comment. The rates of *regular smoking* observed here (6.7% regular smokers) were consistent with national statistics for England showing that regular smoking (at least one cigarette per week) has fallen in recent years (15% in 2009 to 7% in 2016 among 15-year-olds in England; Office for National Statistics, 2018). The lack of a significant effect of the intervention on *regular smoking* may indicate its lack of effectiveness on curbing higher levels of smoking. However, the current study may be lacking in power to detect differences between conditions at such low frequencies of regular smoking (7.2% vs. 6.3% *regular smoking* for control and invention, respectively). Similar arguments apply to breath CO >6 ppm which was low in our sample (1.8% vs. 1.1% for control and invention, respectively).

Nevertheless there are reasons to place reliance on the findings from our outcome of *ever smoking*. The intervention targeted refusals of offers of cigarettes and we assume it will be more effective in relation to not trying that very first cigarette or first few cigarettes than in response to offers of subsequent cigarettes. Our *ever smoking* measure is the best match to this target of preventing the trying of a first cigarette. Our other smoking outcomes (i.e., *any smoking (last 30 days), regular smoking* or *breath CO* >6 ppm) would be less well matched to this target. The present data offer only limited tests of the impact of the intervention on the transition from trying a first cigarette to subsequent smoking or more regular smoking. We did observe significant effects of the intervention on measures of *any smoking* (*last 30 days*) but this may still reflect the early stages (i.e., first few cigarettes) in becoming a regular smoker. Nevertheless, research does suggest a strong relationship between trying one cigarette and progression to becoming a daily smoker (Saddleson et al., 2016; Sargent et al., 2017). For example, a recent meta-analysis (Birge, Duffy, Miler, & Hajek, in 2018) of relevant studies indicated that 68.9% of people who tried one cigarette progressed to daily smoking.

Although systematic reviews indicate mixed evidence for school-based interventions for smoking prevention (Thomas et al., 2015), many countries require the inclusion of some activities for smoking prevention in the curriculum. This being the case, identifying effective school-based interventions (such as the one considered here) can ensure that such activities are worthwhile. Schools are also a valuable setting for smoking prevention because of the consistent access they provide to adolescents from a broad range of backgrounds. School-based interventions such as the present one have the added advantage of being of equivalent effectiveness and reach in different social class/deprivation groups (as shown here for school-based and individual-based measures of deprivation), thus potentially not exacerbating health inequalities. This contrasts with smoking cessation efforts in adults that tend to meet with lower success in lower socioeconomic status groups (Honjo, Tsutsumi, Kawachi, & Kawakami, 2006) and so tend to widen health inequalities.

The current pragmatic cluster randomized controlled trial of an implementation intention-based intervention about how to refuse offers of a cigarette supports the findings of an earlier explanatory trial (Conner & Higgins, 2010) and pilot study (Higgins & Conner, 2003) of the same intervention in United Kingdom adolescents. Together these findings suggest that the repeated formation of implementation intentions of how to refuse offers of cigarettes when combined with antismoking messages may be an effective means to reduce smoking initiation in adolescents. The intervention can be fully implemented by teachers to groups of adolescents in classroom time in any school, thus giving it wide potential reach. The intervention is also uncomplicated and low cost with each implementation requiring a maximum of 30–50 min for adolescents to engage with messages targeting motivation to not smoke and completing an implementation intention sheet. In addition, there was no evidence that the intervention was less effective in groups that tend to show higher rates of smoking (e.g., economically deprived or ethnic minority groups; Drope et al., 2018).

It is worth reiterating that the tested intervention assessed the combined effects of engaging with antismoking motivational messages *and* forming an implementation intention in relation to not smoking. This was consistent with a previous explanatory trial (Conner & Higgins, 2010) showing that this combination was more effective in reducing smoking initiation in adolescents than antismoking messages alone. It is also consistent with theoretical and empirical work showing the effectiveness of implementation intentions to be maximized among those motivated to engage in the behavior (Gollwitzer, 1993; Gollwitzer & Sheeran, 2006).

The current trial tested the effects of forming implementation intentions twice per year over a period of 4 years thus requiring fewer than 7 hr of classroom time per pupil. With these characteristics of significant effects on reducing initiation, wide reach, and low cost the intervention could provide an important means to reduce adolescent smoking initiation. The intervention shows a similar or higher level of effectiveness to school-based peer-led interventions such as the ASSIST program (Campbell et al., 2008) that have been used in a number of schools in the United Kingdom, but at a much lower cost per pupil ($8.64 [£6.40] here vs. $43 [£32] for ASSIST; Hollingworth et al., 2012) and thus, appears substantially more cost-effective ($134–$292 [£99–£205] per smoker avoided here compared with $2,024 [£1,500] reported for ASSIST; Hollingworth et al., 2012). A future article will describe the outcomes from a decision-analytic model incorporating the downstream health benefits and cost savings of smoking prevention that account for current trends in smoking uptake and quit rates and will explore various scenarios of intervention effect attenuation over time.

Key strengths of the current research are retention of the schools throughout the 4-year study and the diversity of the participating schools (e.g., serving pupils from different socioeconomic status groups and different school sizes). The schools were generally representative of schools across the United Kingdom and the two geographical areas they were drawn from in relation to proportion of free school meals. There was some evidence that our schools were slightly more deprived on this measure, although this was only significant in relation to the schools in the Staffordshire area. Although our measures of deprivation were predictive of smoking outcomes, there was no evidence that school or individual measures of deprivation significantly moderated the effectiveness of the intervention. It is also worth noting that the reported intervention effects are for differences between the intervention and control conditions in smoking outcomes taken approximately 6 months after the last intervention session.

There are also a number of weaknesses to the present research. A first weakness is the fact that three control schools withdrew from the study after randomization due to a change in decision to participate by school management. Although the numbers of schools in both conditions at final follow-up exceeded the required numbers based on prior power calculations (Conner et al., 2013), this does not remove the potential for bias that such dropouts can introduce. Relatedly the fact that participants were recruited after randomization could be considered a weakness. A second weakness is the fact that we had to impute measures for a number of participants (particularly in relation to baseline ever smoking which was attributable to the problems of matching data across time points based on a personally generated code). Although it is worth noting that the findings were similar when using nonimputed data. A third weakness was that we were not able to assess longer term effects of our intervention on rates of smoking initiation and whether these differences translate into long-term patterns of regular or irregular smoking or merely delay smoking initiation. A fourth weakness was that we were unable to assess drop-out from the study. Strenuous efforts were made to ensure we tested all eligible adolescents at each school (including running 10 additional sessions at schools when significant numbers of adolescents were not present). However, it remains possible that significant numbers of adolescents were not tested. This might be particularly likely to affect smokers, who may be more likely to be absent from school. However, randomization should ensure that drop-out of smokers did not unduly influence the findings.

In conclusion, the use of repeated implementation intentions about how to refuse the offer of a cigarette plus antismoking messages is an effective and cost-effective intervention to reduce smoking initiation in adolescents. However, impacts on progression to regular smoking remain to be demonstrated.

## Supplementary Material

10.1037/ccp0000387.supp

## Figures and Tables

**Table 1 tbl1:** Descriptive Data for Sample (Comparison of Control and Intervention Conditions)

Measures	Total	Control	Intervention	*p*^1^
Baseline				
School size^2^	940 (305.9)	878.0 (348.0)	990.2 (264.3)	.225
Area				
Leeds	20/45 (44.4%)	8/20 (40.0%)	12/25 (48.0%)	
Staffordshire	25/45 (55.6%)	12/20 (60.0%)	13/25 (52.0%)	.764
Free school meals^2^	16.55 (9.30)	14.97 (6.81)	17.81 (10.87)	.313
Baseline self-reported ever smoking				
Nonsmoker	4,101/4,402 (93.2%)	1,858/1,967 (94.5%)	2,243/2,435 (92.1%)	
Ever smoker	301/4,402 (6.8%)	109/1,967 (5.5%)	192/2,435 (7.9%)	.002
48-month follow-up (baseline never smokers)				
Total *N*	6,155 (100%)	2,719 (100%)	3,436 (100%)	
Gender				
Boys	3,039/6,131 (49.6%)	1,354/2,706 (50.0%)	1,685/3,425 (49.2%)	
Girls	3,092/6,131 (50.4%)	1,352/2,706 (50.0%)	1,740/3,425 (50.8%)	.520
Missing	24	13	11	
Ethnicity				
Non-White	1,038/5,837 (17.8%)	438/2,579 (17.0%)	600/3,258 (18.4%)	
White	4,799/5,837 (82.2%)	2,141/2,681 (83.0%)	2,658/3,258 (81.6%)	.158
Missing	318	140	178	
Family affluence scale^2^	6.24 (1.59)	6.28 (1.57)	6.21 (1.61)	.120
Missing	257	113	144	
Ever smoking				
Nonsmoker	4,051/5,974 (67.8%)	1,700/2,648 (64.2%)	2,351/3,326 (70.7%)	
Ever smoker	1,923/5,974 (32.2%)	948/2,648 (35.8%)	975/3,326 (29.3%)	<.001
Missing	181	71	110	
Any smoking (last 30 days)				
Nonsmoker (0 days)	4,843/5,799 (83.5%)	2,075/2,567 (80.8%)	2,768/3,232 (85.6%)	
Recent smoker (≥1 days)	956/5,799 (16.5%)	492/2,567 (19.2%)	464/3,232 (14.4%)	<.001
Missing	356	152	204	
Regular smoking				
Nonsmoker	5576/5,974 (93.3%)	2,458/2,648 (92.8%)	3,118/3,326 (93.7%)	
Regular smoker	398/5,974 (6.7%)	190/2,648 (7.2%)	208/3,326 (6.3%)	.159
Missing	181	71	110	
Breath CO				
≤6 ppm	5,867/5,951 (98.6%)	2,551/2,599 (98.2%)	3,316/3,352 (98.9%)	
>6 ppm	84/5,951 (1.4%)	48/2,599 (1.8%)	36/3,352 (1.1%)	.014
Missing	204	120	84	
^1^ Difference between intervention and control conditions p-value based on Fisher’s exact test (two-sided). ^2^ Mean and *SD*; *p*-value based on *F*-test on normalized scores.				

**Table 2 tbl2:** Association of Smoking Outcome Measures at Follow-Up With Condition and Demographic Predictors Controlling for Clustering by School Excluding Baseline Smokers Based on Imputed Data (N = 6,155)

	Ever smoking			Any smoking (last 30 days)			Regular smoking			Breath CO >6 ppm		
Predictors	RR	95% CI	*p*	RR	95% CI	*p*	RR	95% CI	*p*	RR	95% CI	*p*
Model 1 without covariates												
Condition												
Control	1.00			1.00			1.00			1.00		
Intervention	0.83	[0.71, 0.97]	.016	0.77	[0.63, 0.93]	.007	0.87	[0.66, 1.13]	.296	0.55	[0.29, 1.04]	.066
Model 2 with covariates												
Condition												
Control	1.00			1.00			1.00			1.00		
Intervention	0.80	[0.70, 0.93]	.003	0.75	[0.62, 0.90]	.002	0.81	[0.64, 1.03]	.092	0.53	[0.31, 0.92]	.024
Free school meals	1.01	[1.001, 1.02]	.022	1.01	[0.997, 1.02]	.159	1.02	[1.003, 1.03]	.016	1.02	[0.99, 1.05]	.306
Gender:												
Boys	1.00			1.00			1.00			1.00		
Girls	1.28	[1.19, 1.37]	<.001	1.01	[0.90, 1.13]	.898	1.02	[0.84, 1.23]	.872	1.06	[0.72, 1.56]	.773
Ethnicity:												
Non-White	1.00			1.00			1.00			1.00		
White	1.17	[1.04, 1.31]	.007	1.01	[0.86, 1.19]	.913	1.38	[1.03, 1.85]	.031	1.19	[0.66, 2.13]	.568
Family Affluence Scale	0.96	[0.94, 0.98]	<.001	0.95	[0.91, 0.98]	.004	0.88	[0.83, 0.93]	<.001	0.81	[0.73, 0.90]	<.001
*Note.* Ever smoking (Model 1: ICC = 0.017; Model 2: ICC = 0.014); any smoking (last 30 days) (ICC = 0.019; Model 2: ICC = 0.015); regular smoking (ICC = 0.033; Model 2: ICC = 0.015); Breath CO > 6 ppm (Model 1: ICC = 0.160; Model 2: ICC = 0.157).
