# Supplementary material for: Effectiveness and Cost-Effectiveness of Repeated Implementation Intention Formation on Adolescent Smoking Initiation: A Cluster Randomized Controlled Trial
Source: J Consult Clin Psychol. 2019 Mar 7;87(5):422–32. doi: 10.1037/ccp0000387 (PMC6474716; doi:10.1037/ccp0000387)
Supplement: Supplementary file 1 [file CCP-2018-1194_Supplementary.docx]

Supplementary Table 1. *Association of ever smoking or regular smoking across all four follow-up time points (2-5) with condition and demographic predictors controlling for clustering by school (N = 6155).*

___________________________________________________________________________________________________________________

Ever Smoking Regular Smoking Breath CO > 6ppm

______________________ ______________________ ______________________

Predictors RR 95% CI *p* RR 95% CI *p* RR 95% CI *p*

___________________________________________________________________________________________________________________

**Model 1 without covariates**

Condition

Control 1·00 1·00 1·00

Intervention 0·85 0·75, 0·97 ·016 0·83 0·64, 1·07 ·156 0·71 0·53, 0·96 ·027

**Model 2 with covariates**

Condition

Control 1·00 1·00 1·00

Intervention 0·83 0·73, 0·94 ·003 0·77 0·61, 0·98 ·036 0·68 0·45, 1·04 ·079

Free school meals 1·01 1·001, 1·01 ·024 1·01 1·003, 1·03 ·019 1·02 0·996, 1·04 ·107

Gender: Boys 1·00 1·00 1·00

Girls 1·19 1·11, 1·27 <·001 1·07 0·90, 1·27 ·462 1·03 0·76, 1·38 ·857

Ethnicity: Non-white 1·00 1·00 1·00

White 1·16 1·05, 1·29 ·003 1·22 0·94, 1·60 ·136 1·19 0·75, 1·90 ·459

Family affluence 0·96 0·94, 0·98 <·001 0·89 0·85, 0·94 <·001 0·84 0·77, 0·91 <·001

___________________________________________________________________________________________________________________

Ever Smoking (step 1: ICC = 0·013; step 2: ICC = 0·010); Regular Smoking (step 1: ICC = 0·038; step 2: ICC = 0·021); Breath CO > 6ppm (step 1: ICC = 0·137; step 2: ICC = 0·123).

Supplementary Table 2. *Costs of the intervention (in UK sterling).*

| **Cost item** | **Mean (per school)*** |
| --- | --- |
| **Base case analysis** |  |
| Material development (printing) costs | **£331.05** |
| Material delivery costs |  |
| Travel time | £67.73 |
| Mileage | £93.37 |
| Training delivery costs |  |
| Travel time | £36.47 |
| Mileage | £50.27 |
| Training delivery time | £28.39 |
| Total delivery | **£276.23** |
| Training receipt costs |  |
| Administration costs in school | £173.58 |
| Attending training session | £303.77 |
| Total receipt | **£477.35** |
| Total undiscounted | £1,084.63 |
| Total discounted | £1,031.04 |
| Total discounted cost per pupil | **£6.44** |
|  |  |
| **Sensitivity analysis (including the sunk costs of intervention design)** |  |
| Researcher time development costs | £348.52 |
| Teacher time for delivering session | £810.05 |
| Grand total undiscounted | £2,243.19 |
| Grand total discounted | £2,132.15 |
| Total discounted cost per pupil | **£13.33** |

*Assumes intervention is received over 4 years; mean 160 school size for per pupil calculation.
